# CCL11 (Eotaxin) Promotes the Advancement of Aging-Related Cardiovascular Diseases

**DOI:** 10.31083/RCM26020

**Published:** 2025-02-13

**Authors:** Tanwei Zhang, Yanhong Huang, Xinmeng Ji, Teng Wu, Pingxi Xiao

**Affiliations:** ^1^Key Laboratory of Targeted Intervention of Cardiovascular Disease and Collaborative Innovation Center for Cardiovascular Translational Medicine, Department of Pathophysiology, Nanjing Medical University, 211166 Nanjing, Jiangsu, China; ^2^Department of Cardiology, The Fourth Affiliated Hospital of Nanjing Medical University, 210031 Nanjing, Jiangsu, China

**Keywords:** CCL11, aging-related diseases, chronic inflammation, cardiovascular diseases

## Abstract

Aging-related diseases, such as cardiovascular diseases (CVDs), neurodegeneration, cancer, etc., have become important factors that threaten the lifespans of older individuals. A chronic inflammatory response is closely related to aging-related diseases. Establishing inflammatory aging clock (iAGE, deep-learning methods on blood immune biomarkers to construct a metric for age-related chronic inflammation) successfully predicted the positive correlation between several factors, including serum C–C-motif chemokine ligand 11 (CCL11) and aging-related diseases. Recently, the role and mechanism of CCL11, an eosinophilic chemokine, in neurodegenerative diseases have been widely reported. Additionally, many research studies have shown a positive correlation with CVDs, but the underlying mechanism remains unknown. This review focuses on the relationship between chronic inflammation and aging. The role of CCL11 will be discussed and summarized in relation to aging-related diseases, especially CVDs.

## 1. Introduction

Aging-associated diseases are those whose incidence positively correlates with age. Most of them are associated with increased levels of cellular senescence and have inflammatory pathogenesis [1]. The global population is aging much faster than previously. According to the World Social Report (previously reported on the World Social Situation), the number of people aged 65 and over is expected to at least double by the middle of the 21st century; thus, the emergence of aging-related diseases will likely also increase, including cardiovascular diseases (CVDs) and neurodegenerative diseases, which alter homeostasis and reduce lifespan as well as life quality [2]. Subsequently, the burden on the public health system will continue to rise, attributable to morbidity and treatment costs.

Studies have demonstrated that cellular senescence, a permanent state of cell cycle arrest induced by cellular stress, is a fundamental aging mechanism contributing to multiple aging-related diseases [3, 4]. Hence, it is essential to clarify the mechanism through which cell senescence occurs and effectively intervene or delay diseases related to later life. Moreover, a persistent chronic inflammatory response is another principal causative factor that leads to aging-related diseases [5]. Researchers have constructed an inflammatory aging clock (iAGE) to identify factors associated with chronic inflammatory responses and successfully predict the positive correlation between cardiovascular aging and masses of immune markers, including C–C-motif chemokine ligand 11 (CCL11) [6].

CCL11, also known as eotaxin-1, is a chemokine belonging to the CC subfamily [7]. Initially described by P.J. Jose in 1994 during experiments on asthma, CCL11 (eotaxin-1) was demonstrated as a potent chemokine that promotes migration and activation of eosinophils participating in the pathogenesis of a broad range of allergy-related diseases [8, 9, 10]. After CCL11 and its homologous receptor C-C motif chemokine receptor 3 (CCR3) were cloned successfully, two other molecules, CCL24 (eotaxin-2) and CCL26 (eotaxin-3), were described according to their similar function of signaling through CCR3, though with low sequence similarity at approximately 30% [11, 12, 13, 14, 15]. These three kinds of eotaxin have similar and distinct functions, whereby all can stimulate eosinophil chemotaxis and exert actions on other innate immune cells [13]. In recent years, CCL11 has been selected as one of the most efficient biomarkers for detecting various aging-related diseases. Growing evidence manifested the promoting effect of CCL11 on aging and aging-related diseases, especially CVDs and neurodegenerative diseases. However, the positive association and specific mechanism between chemokine and diseases are still being studied.

This review will summarize the relationship between cellular senescence, chronic inflammatory response, and aging. Additionally, the effects of CCL11 on multiple aging-related diseases, such as neurodegenerative diseases and CVDs, will be discussed, with an emphasis on the latter. Relevant research will be outlined to demonstrate the correlation function, and a hypothesis that a positive association exists will be proposed. This work will provide an orientation of current research and clarify concrete mechanisms for use in the near future, which will better protect the health of people worldwide and ultimately prolong their lifespan, consequently alleviating the medical and economic pressure caused by aging.

## 2. Cellular Senescence

Cellular senescence is a driving factor of various aging-related diseases [4]. Several lines of evidence suggest that removing senescent cells increases healthy lifespans in murine models [16]. Conversely, research has shown that the accumulation of senescent cells in various tissues contributes to excessive inflammation and an imbalance in tissue homeostasis, leading to diseases later in life, such as cancer, atherosclerosis, and neurodegeneration [3, 17].

### 2.1 Typical Cellular Senescence Process

Cellular senescence is a cell state that limits the proliferative lifespan of cells [18]. Two fundamental mechanisms that result in stable cell cycle arrest have been described: replicative and premature senescence. Replicative senescence is caused by a progressive shortening of telomeres upon each cell division. Additionally, replicative senescence represents physiological responses to prevent genomic instability and, therefore, the accumulation of DNA damage, which can be concluded as cell-intrinsic changes. Premature senescence, independent from telomere shortening, however, is considered a stress response induced by variable intrinsic and extrinsic insults, such as metabolic shock, oxidative and genotoxic stress, mitochondrial dysfunction, oncogenic activation, irradiation, or chemotherapeutic agents [18, 19, 20].

Unlike cell death, senescent cells still maintain metabolic activity for a period of time and show obvious changes, with typical cell cycle arrest, the senescence-associated secretory phenotype (SASP), macromolecular damage, and metabolic disorders [20]. Cellular senescence triggers profound phenotypic changes, expressing and secreting a plethora of soluble and insoluble factors, collectively termed the SASP [21]. The SASP constitutes a combination of bioactive secretions, and common phenotypes include the release of proinflammatory cytokines (interleukin-1 (IL-1), interleukin-6 (IL-6)), chemokines (C-X-C motif chemokine ligand (CXCL) 1, CXCL3), matrix metalloproteinases (MMPs), reactive oxygen species (ROS), growth factors (vascular endothelial growth factor (VEGF), angiogenin), and other signaling molecules secreted by senescent cells [19, 20]. Apart from being a consequence of cellular senescence, SASP also influences the microenvironment, mediating many pathophysiological effects [21].

**Fig. 1.**
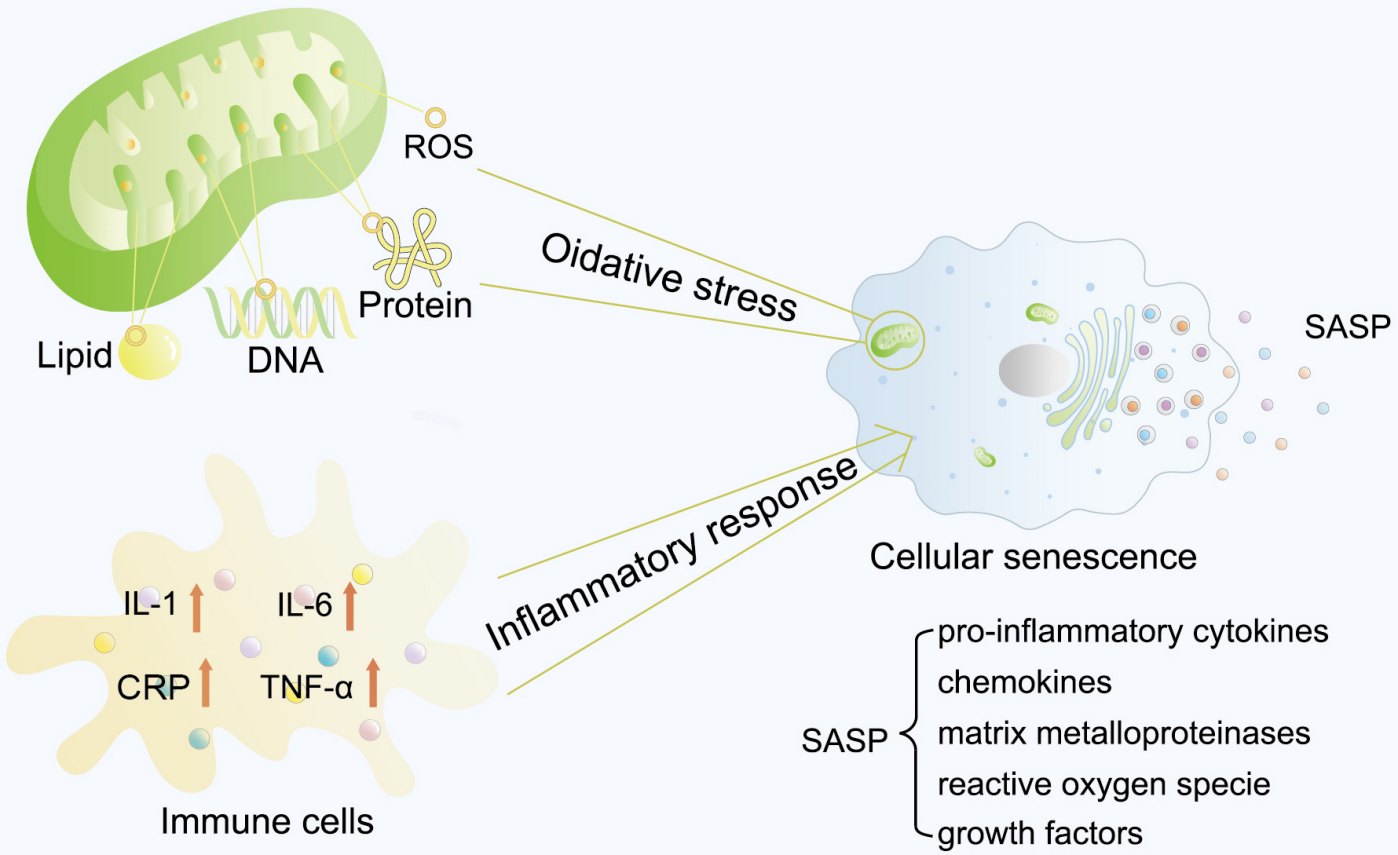
**Typical cellular senescence process**. Excessive oxidative stress and inflammatory response are the two main causes of cell senescence. Mitochondria mainly generate ROS in cells through the electron transport chain. Higher levels of ROS have been reported to severely damage DNA, lipids, and proteins, leading to cellular senescence. Several studies have also reported elevated serum and plasma levels of inflammatory factors in older individuals. These two initiative factors lead to cellular senescence, and the aging cells would thereby express and secret a plethora of soluble and insoluble factors, collectively termed the SASP. ROS, reactive oxygen species; CRP, C-reactive protein; SASP, senescence-associated secretory phenotype; IL-1, interleukin-1; IL-6, interleukin-6; TNF-α, tumor necrosis factor α.

Excessive oxidative stress and the inflammatory response are the two main causes of cell senescence (Fig. 1). ROS, including superoxide anion (O_2_^–^), hydroxyl (HO•), and hydroperoxyl (H_2_O_2_) radicals, are highly potent oxidants containing oxygen [22]. Mainly generated by mitochondria in cells through the electron transport chain, ROS could function as an important mediator in regulating cell growth, cell adhesion, differentiation, and cell death at a properly low level. However, higher ROS levels have been reported to severely damage DNA, lipids, and proteins, leading to cellular senescence [23].

A persistent chronic inflammatory response is another principle that can lead to aging. Based on observational studies of aged organisms, Claudio Franceschi proposed an important hypothesis: older organisms tend to develop a proinflammatory status characterized by high levels of proinflammatory markers in cells and tissues, first termed inflammageing in 2000 [24]. Several studies have also reported elevated serum and plasma levels of inflammatory factors in older individuals, such as IL-1, IL-6, C-reactive protein (CRP), and tumor necrosis factor α (TNF-α) [5, 25, 26, 27]. According to epidemiological studies, inflammageing has been demonstrated as a risk factor for aging-related diseases, such as CVDs, cancer, neurodegenerative diseases, chronic kidney disease, and dementia [28, 29, 30, 31, 32]. Therefore, the successful evaluation or prediction of the level of the chronic inflammatory response can be considered a measurement of our “real age”. More importantly, it can predict or further intervene in various aging-related diseases. Based on the link between inflammation and aging, Buck Institute and Stanford University researchers created an inflammatory aging clock (iAGE) in 2021 to examine multiple immune system biomarkers in the blood and identify predictors of biological aging. These researchers successfully predicted the positive correlation between cardiovascular aging and immune markers, including serum CCL11 [6].

### 2.2 CCL11-Induced Cellular Senescence

**Fig. 2.**
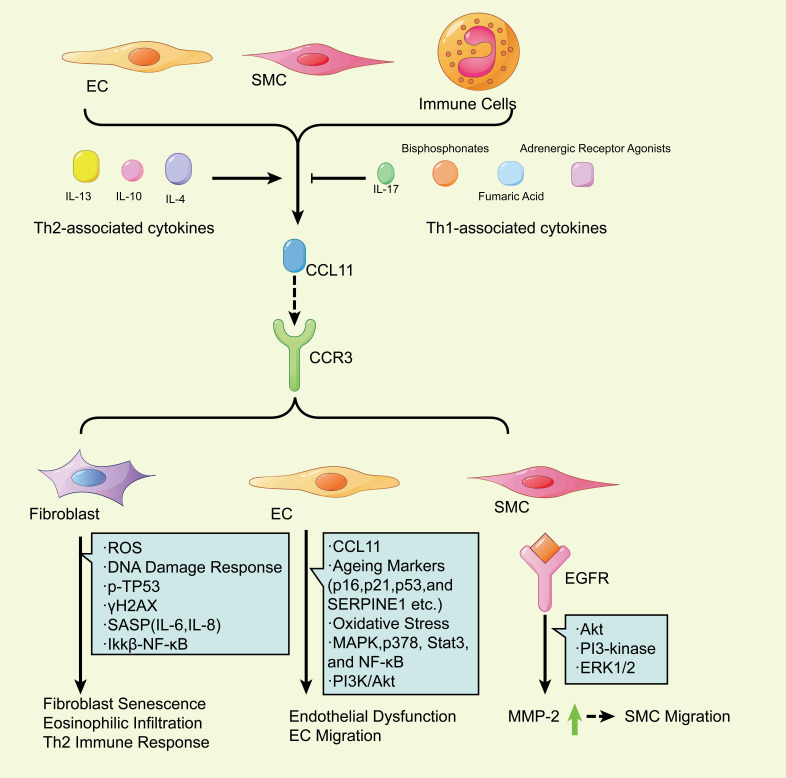
**The mechanisms through which CCL11 regulates cell senescence**. CCL11 could produce SMCs, ECs, and immune cells (eosinophils, macrophages, T cells, and B cells) under the action of Th2-associated cytokines (IL-13, IL-10, and IL-4). Such a process could be suppressed by Th1-associated interferons (IL-17, bisphosphonates, 2 adrenergic receptor agonists, and fumaric acid). CCL11 then binds to CCR3 in various cells and provokes cell senescence. In fibroblasts, CCL11 would induce fibroblast senescence, eosinophilic infiltration, and the Th2 immune response through different pathways, including the DNA damage response, p-TP53 and γH2AX, the SASP (IL-6 and IL-8), and Ikkβ–NF-κB. In ECs, CCL11 would lead to endothelial dysfunction and EC migration via diverse pathways (increased p16, p21, p53, and SERPINE1, oxidative stress, and activation of MAPK p38, Stat3, NF-κB, and PI3K/Akt). In SMCs, CCL11 induces SMC migration through activated PI3-kinase, ERK1/2, and Akt signaling pathways. CCL11, C–C-motif chemokine ligand 11; SMC, smooth muscle cell; EC, endothelial cell; Th2, T helper 2; CCR3, C-C motif chemokine receptor 3; TP53, tumor protein p53; γH2AX, phosphorylated H2A histone family member X; SASP, senescence-associated secretory phenotype; MAPK, mitogen-activated protein kinase; Stat3, signal transducer and activator of transcription 3; PI3, peptidase inhibitor 3; MMP-2, matrix metalloproteinases-2; EGFR, epidermal growth factor receptor; ERK1/2, extracellular regulated kinase 1/2; ROS, reactive oxygen species; Atk, protein kinase B; Ikkβ–NF-κB, inhibitor of nuclear factor kappa B kinase β subunit-nuclear factor-kappa B; SERPINE 1, serpin family E member 1; IL, interleukin.

CCL11 could be produced by many secretory cells (Fig. 2), including smooth muscle cells (SMCs), endothelial cells (ECs), and immune cells (eosinophils, macrophages, T cells, and B cells) under the action of Th2-associated cytokines (IL-13, IL-10 and IL-4) [33, 34]. However, Th1-associated interferons (IL-17, bisphosphonates, 2 adrenergic receptor agonists, and fumaric acid) would suppress such a secretory process [13].

CCL11 has been reported to be involved in numerous cellular senescence. In asthma, the study revealed that eotaxin-1/CCL11 promoted ROS production and increased the activation of the DNA damage response (DDR), p-tumor protein p53 (TP53), and phosphorylated H2A histone family member X (γH2AX) in lung fibroblasts, accompanied by the promotion of cellular senescence and secretion of the SASP, including IL-6 and IL-8 [35]. In a study on human atopic dermatitis (AD), CCL11 was reported to be overexpressed in fibroblasts, causing the fibroblasts to destroy Ikkβ–NF-κB (inhibitor of nuclear factor kappa B kinase β subunit-nuclear factor-kappa B) under homeostasis conditions to abnormally induce skin inflammation, which is characterized by eosinophilic infiltration and a subsequent Th2 immune response [36]. For the epithelial cells, research has shown that airway epithelial lung cells overexpressing CCL11 were accompanied by increasing aging markers such as CDKN2A (p16INK4a), p21, p53, and serpin family E member 1 (SERPINE1) in atopic asthmatic patients [35]. For human coronary artery endothelial cells, CCL11 could increase oxidative stress and activation of mitogen-activated protein kinase (MAPK) p38, signal transducer and activator of transcription 3 (Stat3), and NF-κB, contributing to endothelial dysfunction during vascular lesion formation [37]. Moreover, the CCL11–CCR3 interaction mainly activated the phosphatidylinositol-3-kinase/protein kinase B (PI3K/Akt) signaling pathway in human umbilical vein endothelial cells (HUVECs), promoting endothelial cell migration and inducing weak proliferation [38]. CCL11 is also regarded as a potent chemotactic factor for SMCs, which could induce CCR3-dependent SMC migration [39]. Moreover, CCL11, together with stromal cell-derived factor (SDF), was verified to activate the growth factor receptor (EGFR) to induce matrix metalloproteinases-2 (MMP-2) through different mechanisms: both activated the PI3 kinase, extracellular regulated kinase (ERK) 1/2, and Akt signaling pathways, leading to SMC migration [40].

## 3. Association of CCL11 with Aging-Related Diseases

CCL11 is reported to be associated with the progression of multiple aging-related diseases. Researchers have investigated elevated levels of CCL11 in aging-related diseases and found a role of CCL11 in relevant mechanisms.

### 3.1 CCL11 and Neurodegenerative Diseases

The regulatory effect of CCL11 in neurodegenerative diseases has been widely discussed. Growing evidence has been reviewed regarding the association between CCL11 and multiple neurodegenerative diseases, including Alzheimer’s disease (AD), Huntington’s disease (HD), amyotrophic lateral sclerosis (ALS),multiple sclerosis (MS), secondary progressive multiple sclerosis (SPMS), and chronic traumatic encephalopathy (CTE).

The current increasing incidence of neurodegenerative diseases has caused enormous losses to health and the global economy. Particularly, Alzheimer’s disease, which is the most common neurodegenerative disorder, affects approximately 28 to 38 million people worldwide, followed by HD, ALS, and SPMS [41]. However, developing therapies for these diseases remains limited. Hence, a clear clarification of the neurodegenerative mechanisms involved is required. Recently, several lines have demonstrated that CCL11 and related molecules might contribute to degenerative processes in the central nervous system (CNS) [13].

**Fig. 3.**
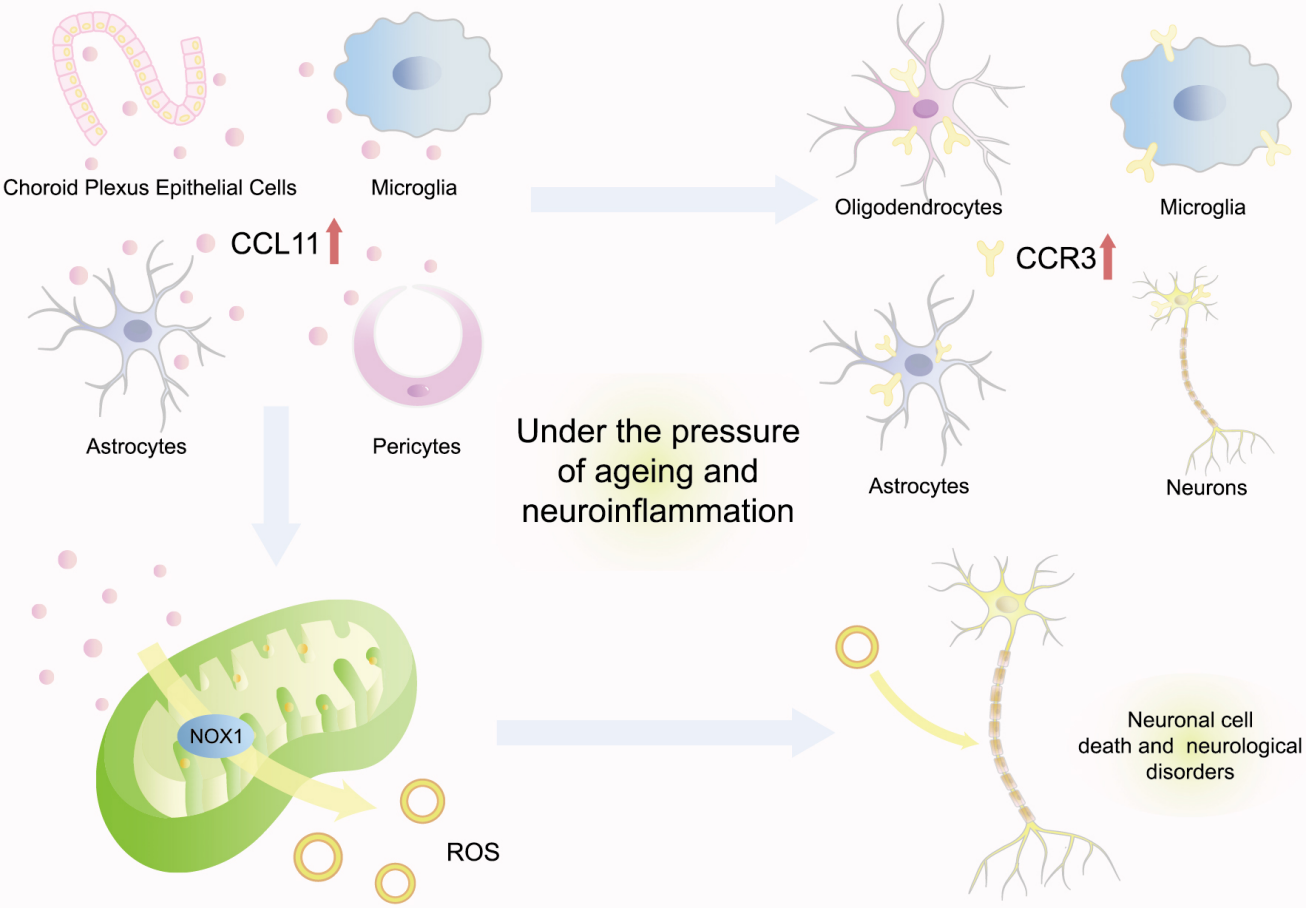
**CCL11 promotes neurodegeneration**. Under the pressure of aging and neuroinflammation, choroid plexus epithelial cells could be provoked to secrete more CCL11 *in vivo* as astrocytes, pericytes, and microglia. CCL11 cognate receptor CCR3 is also highly expressed within the brain by multiple neurocytes. Moreover, CCL11 produces ROS via NOX1 to induce excitotoxic neuronal cell death, which would potentiate the pathogenesis of various neurological disorders. CCL11, C–C-motif chemokine ligand 11; ROS, reactive oxygen species; NOX1, nicotinamide adenine dinucleotide phosphate-oxidase 1; CCR3, C-C motif chemokine receptor 3.

CCL11 has been demonstrated to promote neurodegeneration by enhancing oxidative stress, reducing neurogenesis, and promoting neuroinflammation (Fig. 3). Research has indicated that CCL11 was mainly synthesized by microglia and then transported to the brain through the blood–brain barrier (BBB) [42]. Under the pressure of aging and neuroinflammation, choroid plexus epithelial cells could be provoked to secrete more CCL11 *in vivo* in old mice, such as astrocytes, pericytes, and microglia [13, 42, 43]. Furthermore, CCR3, the cognate receptor for CCL11, was also highly expressed within the brain by multiple varieties of neurocytes, including microglia, oligodendrocytes, astrocytes, and neurons [13]. Bijay Parajuli investigated that activated astrocytes could mainly secrete CCL11 and then promote microglial migration and production of ROS via nicotinamide adenine dinucleotide phosphate-oxidase 1 (NOX1) to induce excitotoxic neuronal cell death, which would potentiate the pathogenesis of various neurological disorders [43]. Moreover, the intensification of neuronal degeneration at the animal level further supported the positive correlation between CCL11 and neuronal senescence. Both young mice intraperitoneally injected with CCL11 and aging mice with naturally increasing CCL11 were detected with a significant decrease in neurogenesis [42]. Cognitive functions were consequently impaired, characterized by cellular changes consistent with markedly decreased adult neurogenesis and increased neuroinflammation, loss in synaptic plasticity, and behavioral deficits in contextual fear conditioning and radial arm water maze paradigms [42]. This evidence suggested a positive association between CCL11 and neurodegeneration.

Recent studies focusing on specific neurodegenerative diseases, such as Alzheimer’s disease, HD, ALS, SPMS, and CTE, also presented similar tendencies, implying an essential role of CCL11 as a potential neuroinflammation biomarker for distinguishing between different neuroinflammation conditions and therapeutic targets in these disorders [44, 45, 46, 47, 48, 49, 50, 51, 52, 53].

CCL11, known for its role in neurodegenerative diseases by promoting neuroinflammation and oxidative damage, is also critical in vascular inflammation and atherosclerosis [40, 54, 55, 56]. These shared pathways suggest that CCL11 contributes to cardiovascular and neurodegenerative diseases through chronic inflammation, highlighting the interconnectedness of systemic aging processes.

Studies have also demonstrated that risk factors for cardiovascular diseases, including atherosclerosis and hypertension, are strongly associated with an increased risk of neurodegenerative diseases such as Alzheimer’s [57, 58, 59]. The role of CCL11 in promoting vascular inflammation may be a key factor linking cardiovascular and neurodegenerative pathologies.

### 3.2 CCL11 and Broader Systemic Aging

In addition to neurodegeneration, CCL11 has also been investigated in broader systemic aging mechanisms, including immune system decline and inflammation. CD4+ regulatory T (Treg) cells play an important role in immune tolerance and antitumor immunosuppression. CCL11 was shown to increase the CD4+CD25+Foxp3+ Treg cells proportion, CCR3 and Foxp3 expression, and the release of IL-2 and transforming growth factor (TGF) β1 in non-tumor-associated CD4+ T cells through the STAT5 signaling pathway [60]. Therapeutic interventions for cancer treatment would cause thymus damage and limit the recovery of protective immunity. These damages could be alleviated through CCR3-dependent colonization, which re-establishes the epithelial microenvironments that control thymopoiesis. Meanwhile, the expression of CCL11 would be triggered by natural killer T (NKT) cells through IL-4 receptor signaling, helping restore thymus function during the re-establishment of the adaptive immune system [61]. In research on the immunomodulatory compounds in cardiovascular disease, CCL11 was selected as a crucial inflammatory immunophenotype [62].

Regarding the effects of CCL11 on chronic inflammation, CCL11 is recognized for its contribution to chronic low-grade inflammation, which accelerates aging across various organ systems. Increased serum levels of CCL11 have been widely detected in multiple diseases with chronic inflammation of the airways, such as asthma and chronic obstructive pulmonary disease (COPD) [34, 35, 63, 64]. Additionally, growing evidence suggests an association between elevated CCL11 and gastrointestinal inflammation [65]. In a study on chronic inflammatory disease with disturbed bone remodeling, the CCL11/CCR3 pathway was also investigated to drive the expression of CCL11 in bone tissue and its novel role in osteoclast migration and resorption, which could be a new target for the treatment of inflammatory bone resorption [66].

Above all, various research studies show the involvement of CCL11 in neurodegeneration and broader systemic aging mechanisms, further suggesting a potential role for this chemokine in the pathogenesis of CVDs.

### 3.3 CCL11 and Cardiovascular Diseases

Despite significant decreases in prevalence over the last three decades, cardiovascular diseases remain the leading causes of morbidity and mortality in developed nations [67]. Further, aging is by far the strongest independent cardiovascular risk factor that dwarfs the impact of traditional risk factors, with 90% of all cardiovascular diseases occurring in adults aged 40 and older [67, 68].

Cardiovascular aging is a structural and fundamental degenerative alteration, with blood vessels gradually losing their original function with age. As a special type of organ senescence, cardiovascular aging is characterized by structural and functional vascular impairments, such as increased arterial stiffness and decreased compliance, which has deleterious effects on tissue oxygenation, nutrient delivery, and waste removal and, thus, negatively affects multiple organ functions [69]. At the cellular level, cardiovascular aging is mainly manifested by the morphological changes occurring on ECs in the intima and vascular SMCs in the mesa, as ECs and SMCs are critical building blocks of blood vessels and play dominant roles in age-induced cardiovascular dysfunction [2].

Apart from the intrinsic factors such as the phenotypic switch of ECs and SMCs, various extrinsic alterations caused by diseases and changes in cell–cell and cell–matrix interactions also promote vascular aging. Common extrinsic factors include atherosclerosis, hypertension, chronic inflammation, vascular wall stiffness, and vascular cell communication [2].

As an important group of aging-related diseases, cardiovascular diseases are also closely related to chronic inflammation. Epidemiological studies have found that inflammageing is a risk factor for cardiovascular diseases [5]. The characteristic targeting of relevant processes in experimental models has been shown to attenuate CVDs such as myocardial infarction and to predict cardiovascular events in various studies [28, 70].

**Fig. 4.**
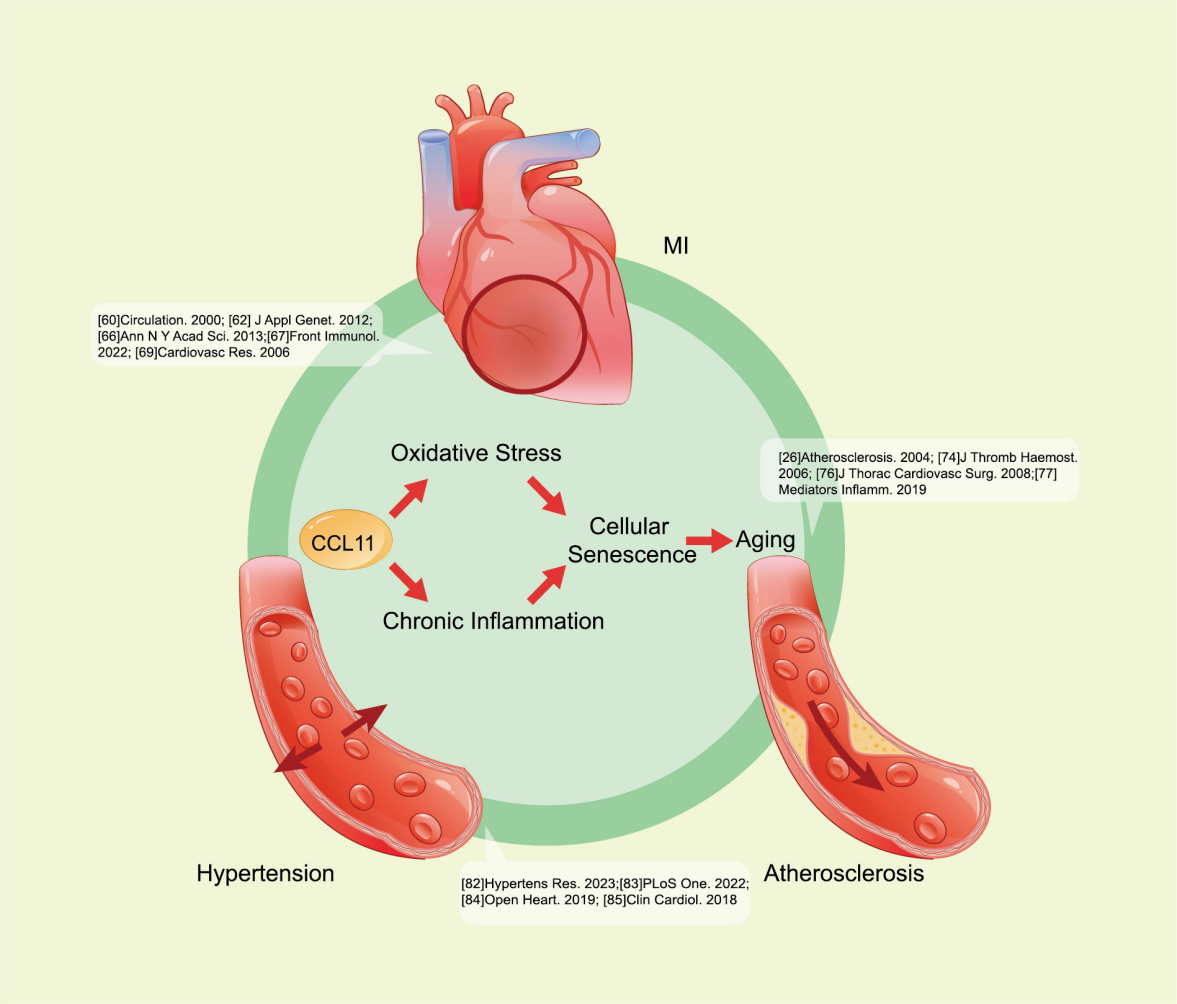
**CCL11 promotes cardiovascular disease progression**. CCL11 contributes to oxidative stress and chronic inflammation, then leads to cellular senescence, and further promotes CVDs, such as atherosclerosis, MI, and hypertension. The specific pathways are as follows: CCL11 downregulates tight junction proteins, increases oxidative stress, and activates MAPK p38, Stat3, and NF-κB in ECs of coronary arteries, causing endothelial dysfunction. CCL11 induces proMMP-2 activation of the EGFR, leading to SMC senescence. CCL11 may also directly contribute to angiogenesis. All of these outcomes are critical factors to atherosclerosis and other cardiovascular diseases, suggesting a role for CCL11 in promoting cardiovascular disease progression. CCL11, C–C-motif chemokine ligand 11; MI, myocardial infarction; CVDs, cardiovascular diseases; proMMP-2, pro-matrix metalloproteinases-2; EGFR, epidermal growth factor receptor; SMC, smooth muscle cell; MAPK, mitogen-activated protein kinase; NF-κB, nuclear factor kappa B; Stat3, signal transducer and activator of transcription 3; ECs, endothelial cells.

Except for the neurodegenerative diseases mentioned above, the role of CCL11 in the pathogenesis of cardiovascular diseases remains a research priority (Fig. 4). The relevance of CCL11 to cardiovascular disease risk has been well established.

Regarding the mechanism through which abnormally elevated CCL11 affects vascular aging, studies showed that CCL11 contributes to chronic inflammation and cellular senescence in cardiovascular aging [37, 40, 71, 72]. CCL11 significantly increases vascular permeability through the downregulation of tight junction proteins, an increase in oxidative stress, and activation of MAPK p38, Stat3, and NF-κB in coronary artery endothelial cells, exerting a crucial impact on endothelial dysfunction during vascular lesion formation [37]. Research showed that MMP-2 is a member of the MMP family critical to SMC migration. As the regulator of proMMP-2 expression and by engaging in receptor cross-talk, CCL11 could induce proMMP-2 activation of the EGFR together with SDF, indicating its critical role in atherosclerosis, restenosis, and plaque rupturing [40]. Thus, CCL11 may directly contribute to angiogenesis [71]. Moreover, CCL11 has been selected as a typical proinflammatory phenotype in human vascular smooth muscle cells [72]. These lines have demonstrated that elevated CCL11 correlates with impaired angiogenesis and endothelial dysfunction, which are critical to atherosclerosis and other cardiovascular diseases.

#### 3.3.1 Atherosclerosis

Atherosclerosis is a chronic inflammatory disease occurring in large and medium-sized arteries and secondarily causes CVDs, including ischemic heart disease, strokes, and peripheral vascular disease [73]. Presently, atherosclerotic cardiovascular diseases account for the majority of mortality worldwide, with the major pool of risk in not only Western countries but also the more populated developing world [74]. The morbidity of atherosclerosis requires elevated LDL (low-density lipoprotein) cholesterol, the most abundant atherogenic lipoprotein in plasma [75]. As the deliverer of cholesterol to the artery wall, LDL contributes to varying degrees of atherosclerotic diseases according to the corresponding duration and extent of exposure if the concentrations are above ideal [74]. In addition to dyslipidemia, inflammation is essential in advancing atherogenesis progress, providing pathways linking lipids and other traditional risk factors [74]. Moreover, biomarker studies have detected increased inflammation indicators in cardiovascular disease [74]. Hence, cardiovascular diseases could be found at early stages, and the course of the disease could be classified if powerful biomarkers could be identified.

Overexpression of the *CCL11* and *CCR3* genes has been demonstrated in human atherosclerosis. In research by Kathleen J Haley, CCL11 was reported to be predominantly expressed by SMCs, playing a part in atherosclerotic vascular inflammation on the one hand. Alternatively, CCR3, the receptor for CCL11, could be induced in several cell types, including macrophages and mast cells, except for in eosinophils, where CCL11 is constitutively expressed [76]. In patients with coronary atherosclerosis, concentrations of eotaxin, the eosinophil-specific chemoattraction, were also reported to be elevated in the inflammation and pathogenesis, regulating eosinophil accumulation through its effect on the adhesion molecules on microvascular endothelial cells [77].

Moreover, the polymorphism of the *CCL11* gene could also influence the development and pathological process of CVD. A substitution of T for A at amino acid 23 in the *eotaxin* gene was reported to be associated with an increased risk for incident myocardial infarction, supporting the emerging hypothesis that eotaxin participated in atherosclerosis [9]. The (GAAGGA) (n) hexanucleotide was associated with the severity of CVDs, and the 67 GG was associated with an acute form of CVDs, thus functioning as a novel biomarker of CVDs [56]. To date, many researchers have chosen CCL11 as one of the characteristic biomarkers of atherosclerosis and developed a risk assessment model based on the concentrations of a series of cytokines and chemokines, including CCL11. In 2007, a combination of circulating chemokines was proven to accurately distinguish individuals with clinically significant CVD from those with no prior history of CVD [78]. The CHD Risk Assessment (CHDRA) model, based on serum protein biomarkers, including CCL11, was developed to assess the risk of acute cardiovascular events [79, 80]. For individuals currently assessed with an intermediate risk, this prognostic algorithm provides a remarkable incremental benefit over using clinical risk factors alone [77]. In many other studies, CCL11 has also been chosen as one of the indispensable biomarkers of atherosclerosis to evaluate the effect of pharmacotherapy on atherosclerosis or to investigate the detailed mechanism of atherosclerosis [40, 54, 62, 72, 81].

It seems that the positive correlation between CCL11 and atherosclerosis progress has been universally recognized, and several hypotheses on the underlying mechanism of this association have been proposed. However, a detailed clarification of such association, especially intracellular mechanism and downstream effector proteins, remains unknown and awaits further investigation.

#### 3.3.2 Myocardial Infarction (MI)

Generally speaking, acute myocardial infarction (AMI) is described as a heart attack caused by a decrease or stoppage of blood flow to a portion of the heart and the subsequent necrosis of heart muscle. Mostly, this necrosis is triggered by a blood clot in the epicardial artery, and atherosclerosis with subsequent inflammation is the most common and vital driver of thrombosis [82]. Epidemiologically, the incidence of myocardial infarction can be termed as a substitution for the prevalence of coronary artery disease in a certain population [83]. From thrombolytic therapy to dissolve intracoronary thrombus and percutaneous coronary intervention (PCI) to reperfusion therapy, the treatment of AMI has been developed for years [82]. However, the mortality of patients with cardiogenic shock remains high at over 40% within 30 days [82]. In addition to those therapies as described above, earlier intervention is more severely required to reduce the mortality in coronary artery disease radically, and multiple risk factors are proposed to be attached with more importance and attention [84].

The single-nucleotide polymorphisms (SNPs) and overexpression of the *CCL11* gene have been reported to be associated with AMI. Research has manifested that threonine for alanine substitution in the *CCL11* gene was related to the morbidity of AMI, and homozygous carriers of the *T23* allele were reported to be at increased risk of myocardial infarction, showing that this polymorphism could be useful as a marker for risk assessment [9, 85]. Furthermore, eotaxin-1, troponin-I, creatine kinase (CK), and creatine kinase MB (CKMB) levels were detected using an enzyme-linked immunosorbent assay (ELISA) and were statistically elevated among 42 patients diagnosed with AMI compared to those among 40 healthy controls [86]. CCR3-mediated interactions are demonstrated to regulate the endogenous migration of CD34+ progenitors from bone marrow to ischemic myocardium, resulting in therapeutic neovascularization for tissue repair after AMI [87]. Moreover, in a closed-chest acute murine MI/R model, CCL11 protein levels, one of the CD4+ T cell-associated chemokines, was elevated on day 7 and at day 14 in heart tissues but lower on day 14 compared with day 7, suggesting that CCL11 might participate in the cardiac repairing and remodeling after AMI/R [88]. According to the above data, the participation of CCL11 in myocardial infarction has been justified, but how CCL11 contributes to the pathogenesis of myocardial infarction will need additional investigation.

#### 3.3.3 Hypertension

Hypertension, characterized by persistent systolic blood pressure (SBP), is a major public health concern, affecting 1.13 billion adults worldwide [89]. Hypertension is a leading cause of CVD events and death. From lifestyle modifications (weight loss, dietary, limited alcohol consumption, sodium reduction, and potassium supplementation, etc.) to pharmaceutical therapies (thiazide or thiazide-like diuretics, angiotensin-converting enzyme inhibitors or angiotensin receptor blockers, calcium channel blockers, etc.), considerable advances have been made in antihypertensive treatments, while the prevalence of hypertension has continued to increase over the past 40 years [90, 91]. As a result, further studies are warranted to develop prevention and control processes for hypertension.

Several lines have demonstrated the relationship between CCL11 and hypertension. Based on the analysis of CCL11 polymorphism in the Xinjiang Han population, six different CCL11 gene polymorphism phenotypes were found, and the correlation between CCL11 gene polymorphism and the risk of atherosclerosis and hypertension was confirmed [92]. Through applying machine-learning models, a panel of protein markers was found to identify hypertensive disorders of pregnancy (HDP). CCL11 was included and showed alterations in the disease group compared to healthy pregnant controls [93]. Research on the association between second-trimester cytokine profiles and HDP or the association between lower serum vitamin D metabolite and circulating chemokines in women with HDP also presented similar answers [94]. Moreover, CCL11 has been selected in developing a clinical and proteomics multiple-marker scoring strategy to diagnose obstructive peripheral arterial disease (PAD), and higher concentrations of CCL11 were notably detected in patients with severe PAD (history of hypertension) [95, 96]. Above all, the relevance of CCL11 in multiple hypertension processes has been proven. Nevertheless, a clear clarification of the definite association between these two objects has yet to be proposed, and the mechanism underlying such a relationship remains to be revealed.

## 4. Discussion

This review summarizes the association between eosinophilic chemokine CCL11 with aging-related diseases, including neurodegenerative diseases and CVDs. Although this study mainly focuses on the positive correlation, it also highlights the differential regulation of CCL11 in various diseases. Although CCL11 is generally associated with neuroinflammation in neurodegenerative diseases, a comparative study showed that CCL11 was increased in the CNS of CTE but not in AD [53]. Furthermore, David E. Mosedale reported that there was no difference in the levels of circulating CCL1 between subjects with and without atherosclerosis. However, a transient increase in circulating chemokine levels following AMI might exist [97]. These divergent results may be attributed to differences in study design, detection method, or stages of disease progression being investigated. Meanwhile, CCL11 concentration could also be regulated by changes in cytokines and signaling pathways specific to certain disease processes.

Regarding the treatment of aging-related diseases, CCL11 has emerged as a potential target, and experimental models have shown that neutralizing CCL11 might reduce proinflammatory markers and improve disease outcomes. Previous research has proven that anti-CCL11 neutralizing antibodies could reduce the production of proinflammatory factors as well as the CD4 +/CD8 + T cells infiltration in the substantia nigra of mice, hence improving motor symptoms in PD mice [98]. Additionally, in animal models, neutralization of CCL11 may prevent nigrostriatal neurodegeneration, alleviating PD progression [99]. In standard-reared aged mice, treatment with an anti-ccl11 antibody led to environmental enrichment-like improvements in spatial memory, hippocampal neurogenesis, and microglial activation [100]. Moreover, possible novel treatments targeting CCL-11 have been proposed. The increasing production of CCL-11 could be attenuated by glucocorticoids, minocycline, resveratrol, and anti-CCL11 antibodies [99]. However, anti-CCL11 therapy in cardiovascular diseases has made little progress and needs further research.

## 5. Conclusions

This article summarized the correlation between inflammatory response and aging-related diseases and emphasized the role of CCL11 in various aging-related diseases, especially CVDs. Owing to positive correlations, CCL11 has become one of the essential biological targets for detecting these diseases and has manifested differential regulation in different diseases or stages of disease progression, indicating its potential to identify diverse aging-related diseases. Concurrently, a novel therapeutic strategy, anti-CCL11 therapy, was proposed and suggested for neurodegenerative and cardiovascular diseases. In the study of neurodegenerative diseases, CCL11 neutralization has shown feasibility in animal models. However, the effect of this treatment on cardiovascular disease is reduced and needs further investigation. In addition, clinical trials for CCL11 are currently vacant, meaning the efficacy and safety of anti-CCL11 therapy in humans cannot be guaranteed.

The specific molecular mechanism through which CCL11 promotes vascular cell senescence and further aggravates aging-related CVDs remains unclear. Many researchers have conducted studies for particular diseases, but a system has yet to be formed. The downstream signaling pathway of CCL11 in promoting cell aging is also vacant. Thus, to develop newer and improved treatment methods for aging-related diseases around CCL11, it is urgent to explore and complete the mechanism of CCL11 regulating aging-related diseases, especially CVDs.

We expect future studies will soon fill these gaps and contribute to preventing and treating aging-related cardiovascular diseases.

## References

[1] Borghesan M, Hoogaars WMH, Varela-Eirin M, Talma N, Demaria M (2020). A Senescence-Centric View of Aging: Implications for Longevity and Disease. *Trends in Cell Biology*.

[2] Mistriotis P, Andreadis ST (2017). Vascular aging: Molecular mechanisms and potential treatments for vascular rejuvenation. *Ageing Research Reviews*.

[3] Childs BG, Gluscevic M, Baker DJ, Laberge RM, Marquess D, Dananberg J (2017). Senescent cells: an emerging target for diseases of ageing. *Nature Reviews. Drug Discovery*.

[4] Guo J, Huang X, Dou L, Yan M, Shen T, Tang W (2022). Aging and aging-related diseases: from molecular mechanisms to interventions and treatments. *Signal Transduction and Targeted Therapy*.

[5] Ferrucci L, Fabbri E (2018). Inflammageing: chronic inflammation in ageing, cardiovascular disease, and frailty. *Nature Reviews. Cardiology*.

[6] Sayed N, Huang Y, Nguyen K, Krejciova-Rajaniemi Z, Grawe AP, Gao T (2021). An inflammatory aging clock (iAge) based on deep learning tracks multimorbidity, immunosenescence, frailty and cardiovascular aging. *Nature Aging*.

[7] Tay HL, Foster PS (2020). Biologics or immunotherapeutics for asthma?. *Pharmacological Research*.

[8] Jose PJ, Adcock IM, Griffiths-Johnson DA, Berkman N, Wells TN, Williams TJ (1994). Eotaxin: cloning of an eosinophil chemoattractant cytokine and increased mRNA expression in allergen-challenged guinea-pig lungs. *Biochemical and Biophysical Research Communications*.

[9] Zee RYL, Cook NR, Cheng S, Erlich HA, Lindpaintner K, Lee RT (2004). Threonine for alanine substitution in the eotaxin (CCL11) gene and the risk of incident myocardial infarction. *Atherosclerosis*.

[10] Raghuraman G, Hsiung J, Zuniga MC, Baughman BD, Hitchner E, Guzman RJ (2017). Eotaxin Augments Calcification in Vascular Smooth Muscle Cells. *Journal of Cellular Biochemistry*.

[11] Ponath PD, Qin S, Post TW, Wang J, Wu L, Gerard NP (1996). Molecular cloning and characterization of a human eotaxin receptor expressed selectively on eosinophils. *The Journal of Experimental Medicine*.

[12] Ponath PD, Qin S, Ringler DJ, Clark-Lewis I, Wang J, Kassam N (1996). Cloning of the human eosinophil chemoattractant, eotaxin. Expression, receptor binding, and functional properties suggest a mechanism for the selective recruitment of eosinophils. *The Journal of Clinical Investigation*.

[13] Huber AK, Giles DA, Segal BM, Irani DN (2018). An emerging role for eotaxins in neurodegenerative disease. *Clinical Immunology (Orlando, Fla.)*.

[14] Forssmann U, Uguccioni M, Loetscher P, Dahinden CA, Langen H, Thelen M (1997). Eotaxin-2, a novel CC chemokine that is selective for the chemokine receptor CCR3, and acts like eotaxin on human eosinophil and basophil leukocytes. *The Journal of Experimental Medicine*.

[15] Kitaura M, Suzuki N, Imai T, Takagi S, Suzuki R, Nakajima T (1999). Molecular cloning of a novel human CC chemokine (Eotaxin-3) that is a functional ligand of CC chemokine receptor 3. *The Journal of Biological Chemistry*.

[16] Baker DJ, Childs BG, Durik M, Wijers ME, Sieben CJ, Zhong J (2016). Naturally occurring p16(Ink4a)-positive cells shorten healthy lifespan. *Nature*.

[17] Wang TW, Johmura Y, Suzuki N, Omori S, Migita T, Yamaguchi K (2022). Blocking PD-L1-PD-1 improves senescence surveillance and ageing phenotypes. *Nature*.

[18] Calcinotto A, Kohli J, Zagato E, Pellegrini L, Demaria M, Alimonti A (2019). Cellular Senescence: Aging, Cancer, and Injury. *Physiological Reviews*.

[19] Herranz N, Gil J (2018). Mechanisms and functions of cellular senescence. *The Journal of Clinical Investigation*.

[20] Gorgoulis V, Adams PD, Alimonti A, Bennett DC, Bischof O, Bishop C (2019). Cellular Senescence: Defining a Path Forward. *Cell*.

[21] Birch J, Gil J (2020). Senescence and the SASP: many therapeutic avenues. *Genes & Development*.

[22] Zorov DB, Juhaszova M, Sollott SJ (2014). Mitochondrial reactive oxygen species (ROS) and ROS-induced ROS release. *Physiological Reviews*.

[23] Chelombitko MA (2018). Role of reactive oxygen species in inflammation: a minireview. *Moscow University Biological Sciences Bulletin*.

[24] Franceschi C, Bonafè M, Valensin S, Olivieri F, De Luca M, Ottaviani E (2000). Inflamm-aging. An evolutionary perspective on immunosenescence. *Annals of the New York Academy of Sciences*.

[25] Gerli R, Monti D, Bistoni O, Mazzone AM, Peri G, Cossarizza A (2000). Chemokines, sTNF-Rs and sCD30 serum levels in healthy aged people and centenarians. *Mechanisms of Ageing and Development*.

[26] Newman AB, Sanders JL, Kizer JR, Boudreau RM, Odden MC, Zeki Al Hazzouri A (2016). Trajectories of function and biomarkers with age: the CHS All Stars Study. *International Journal of Epidemiology*.

[27] Ferrucci L, Semba RD, Guralnik JM, Ershler WB, Bandinelli S, Patel KV (2010). Proinflammatory state, hepcidin, and anemia in older persons. *Blood*.

[28] Ruparelia N, Chai JT, Fisher EA, Choudhury RP (2017). Inflammatory processes in cardiovascular disease: a route to targeted therapies. *Nature Reviews. Cardiology*.

[29] Leonardi GC, Accardi G, Monastero R, Nicoletti F, Libra M (2018). Ageing: from inflammation to cancer. *Immunity & Ageing: i & a*.

[30] Salimi S, Shardell MD, Seliger SL, Bandinelli S, Guralnik JM, Ferrucci L (2018). Inflammation and Trajectory of Renal Function in Community-Dwelling Older Adults. *Journal of the American Geriatrics Society*.

[31] Gorelick PB (2010). Role of inflammation in cognitive impairment: results of observational epidemiological studies and clinical trials. *Annals of the New York Academy of Sciences*.

[32] Lu Y, Jarrahi A, Moore N, Bartoli M, Brann DW, Baban B (2023). Inflammaging, cellular senescence, and cognitive aging after traumatic brain injury. *Neurobiology of Disease*.

[33] Fu CL, Ye YL, Lee YL, Chiang BL (2006). Effects of overexpression of IL-10, IL-12, TGF-beta and IL-4 on allergen induced change in bronchial responsiveness. *Respiratory Research*.

[34] Lv J, Xiong Y, Li W, Cui X, Cheng X, Leng Q (2018). IL-37 inhibits IL-4/IL-13-induced CCL11 production and lung eosinophilia in murine allergic asthma. *Allergy*.

[35] Lavandoski P, Pierdoná V, Maurmann RM, Grun LK, Guma FTCR, Barbé-Tuana FM (2023). Eotaxin-1/CCL11 promotes cellular senescence in human-derived fibroblasts through pro-oxidant and pro-inflammatory pathways. *Frontiers in Immunology*.

[36] Ko KI, Merlet JJ, DerGarabedian BP, Zhen H, Suzuki-Horiuchi Y, Hedberg ML (2022). NF-κB perturbation reveals unique immunomodulatory functions in Prx1+ fibroblasts that promote development of atopic dermatitis. *Science Translational Medicine*.

[37] Jamaluddin MS, Wang X, Wang H, Rafael C, Yao Q, Chen C (2009). Eotaxin increases monolayer permeability of human coronary artery endothelial cells. *Arteriosclerosis, Thrombosis, and Vascular Biology*.

[38] Park JY, Kang YW, Choi BY, Yang YC, Cho BP, Cho WG (2017). CCL11 promotes angiogenic activity by activating the PI3K/Akt pathway in HUVECs. *Journal of Receptor and Signal Transduction Research*.

[39] Kodali RB, Kim WJH, Galaria II, Miller C, Schecter AD, Lira SA (2004). CCL11 (Eotaxin) induces CCR3-dependent smooth muscle cell migration. *Arteriosclerosis, Thrombosis, and Vascular Biology*.

[40] Kodali R, Hajjou M, Berman AB, Bansal MB, Zhang S, Pan JJ (2006). Chemokines induce matrix metalloproteinase-2 through activation of epidermal growth factor receptor in arterial smooth muscle cells. *Cardiovascular Research*.

[41] Katsnelson A, De Strooper B, Zoghbi HY (2016). Neurodegeneration: From cellular concepts to clinical applications. *Science Translational Medicine*.

[42] Villeda SA, Luo J, Mosher KI, Zou B, Britschgi M, Bieri G (2011). The ageing systemic milieu negatively regulates neurogenesis and cognitive function. *Nature*.

[43] Parajuli B, Horiuchi H, Mizuno T, Takeuchi H, Suzumura A (2015). CCL11 enhances excitotoxic neuronal death by producing reactive oxygen species in microglia. *Glia*.

[44] Leung R, Proitsi P, Simmons A, Lunnon K, Güntert A, Kronenberg D (2013). Inflammatory proteins in plasma are associated with severity of Alzheimer’s disease. *PloS One*.

[45] Choi C, Jeong JH, Jang JS, Choi K, Lee J, Kwon J (2008). Multiplex analysis of cytokines in the serum and cerebrospinal fluid of patients with Alzheimer’s disease by color-coded bead technology. *Journal of Clinical Neurology (Seoul, Korea)*.

[46] Wild E, Magnusson A, Lahiri N, Krus U, Orth M, Tabrizi SJ (2011). Abnormal peripheral chemokine profile in Huntington’s disease. *PLoS Currents*.

[47] Furukawa T, Matsui N, Fujita K, Nodera H, Shimizu F, Miyamoto K (2015). CSF cytokine profile distinguishes multifocal motor neuropathy from progressive muscular atrophy. *Neurology(R) Neuroimmunology & Neuroinflammation*.

[48] Kuhle J, Lindberg RLP, Regeniter A, Mehling M, Steck AJ, Kappos L (2009). Increased levels of inflammatory chemokines in amyotrophic lateral sclerosis. *European Journal of Neurology*.

[49] Huber AK, Wang L, Han P, Zhang X, Ekholm S, Srinivasan A (2014). Dysregulation of the IL-23/IL-17 axis and myeloid factors in secondary progressive MS. *Neurology*.

[50] Trapp BD, Nave KA (2008). Multiple sclerosis: an immune or neurodegenerative disorder?. *Annual Review of Neuroscience*.

[51] Tejera-Alhambra M, Casrouge A, de Andrés C, Seyfferth A, Ramos-Medina R, Alonso B (2015). Plasma biomarkers discriminate clinical forms of multiple sclerosis. *PloS One*.

[52] Michael BD, Elsone L, Griffiths MJ, Faragher B, Borrow R, Solomon T (2013). Post-acute serum eosinophil and neutrophil-associated cytokine/chemokine profile can distinguish between patients with neuromyelitis optica and multiple sclerosis; and identifies potential pathophysiological mechanisms - a pilot study. *Cytokine*.

[53] Cherry JD, Stein TD, Tripodis Y, Alvarez VE, Huber BR, Au R (2017). CCL11 is increased in the CNS in chronic traumatic encephalopathy but not in Alzheimer’s disease. *PloS One*.

[54] Lu YJ, Jan YJ, Ko BS, Liang SM, Chen L, Wu CC (2020). Expression of Nik-related kinase in smooth muscle cells attenuates vascular inflammation and intimal hyperplasia. *Aging*.

[55] Weng M, Baron DM, Bloch KD, Luster AD, Lee JJ, Medoff BD (2011). Eosinophils are necessary for pulmonary arterial remodeling in a mouse model of eosinophilic inflammation-induced pulmonary hypertension. *American Journal of Physiology. Lung Cellular and Molecular Physiology*.

[56] Máchal J, Vašků A, Kincl V, Hlavna M, Bartáková V, Jurajda M (2012). Association between three single nucleotide polymorphisms in eotaxin (CCL 11) gene, hexanucleotide repetition upstream, severity and course of coronary atherosclerosis. *Journal of Applied Genetics*.

[57] Cortes-Canteli M, Iadecola C (2020). Alzheimer’s Disease and Vascular Aging: JACC Focus Seminar. *Journal of the American College of Cardiology*.

[58] Saeed A, Lopez O, Cohen A, Reis SE (2023). Cardiovascular Disease and Alzheimer’s Disease: The Heart-Brain Axis. *Journal of the American Heart Association*.

[59] Silva MVF, Loures CDMG, Alves LCV, de Souza LC, Borges KBG, Carvalho MDG (2019). Alzheimer’s disease: risk factors and potentially protective measures. *Journal of Biomedical Science*.

[60] Wang R, Huang K (2020). CCL11 increases the proportion of CD4+CD25+Foxp3+ Treg cells and the production of IL 2 and TGF β by CD4+ T cells via the STAT5 signaling pathway. *Molecular Medicine Reports*.

[61] Cosway EJ, White AJ, Parnell SM, Schweighoffer E, Jolin HE, Bacon A (2022). Eosinophils are an essential element of a type 2 immune axis that controls thymus regeneration. *Science Immunology*.

[62] Grievink HW, Smit V, Huisman BW, Gal P, Yavuz Y, Klerks C (2022). Cardiovascular risk factors: The effects of ageing and smoking on the immune system, an observational clinical study. *Frontiers in Immunology*.

[63] Daldegan MB, Teixeira MM, Talvani A (2005). Concentration of CCL11, CXCL8 and TNF-alpha in sputum and plasma of patients undergoing asthma or chronic obstructive pulmonary disease exacerbation. *Brazilian Journal of Medical and Biological Research*.

[64] Fourtounis J, Wang IM, Mathieu MC, Claveau D, Loo T, Jackson AL (2012). Gene expression profiling following NRF2 and KEAP1 siRNA knockdown in human lung fibroblasts identifies CCL11/Eotaxin-1 as a novel NRF2 regulated gene. *Respiratory Research*.

[65] Adar T, Shteingart S, Ben Ya’acov A, Bar-Gil Shitrit A, Goldin E (2014). From airway inflammation to inflammatory bowel disease: eotaxin-1, a key regulator of intestinal inflammation. *Clinical Immunology (Orlando, Fla.)*.

[66] Kindstedt E, Holm CK, Sulniute R, Martinez-Carrasco I, Lundmark R, Lundberg P (2017). CCL11, a novel mediator of inflammatory bone resorption. *Scientific Reports*.

[67] Seals DR, Alexander LM (2018). Vascular aging. *Journal of Applied Physiology (Bethesda, Md.: 1985)*.

[68] Ungvari Z, Tarantini S, Sorond F, Merkely B, Csiszar A (2020). Mechanisms of Vascular Aging, A Geroscience Perspective: JACC Focus Seminar. *Journal of the American College of Cardiology*.

[69] Ungvari Z, Tarantini S, Donato AJ, Galvan V, Csiszar A (2018). Mechanisms of Vascular Aging. *Circulation Research*.

[70] Siegel D, Devaraj S, Mitra A, Raychaudhuri SP, Raychaudhuri SK, Jialal I (2013). Inflammation, atherosclerosis, and psoriasis. *Clinical Reviews in Allergy & Immunology*.

[71] Salcedo R, Young HA, Ponce ML, Ward JM, Kleinman HK, Murphy WJ (2001). Eotaxin (CCL11) induces in vivo angiogenic responses by human CCR3+ endothelial cells. *Journal of Immunology (Baltimore, Md.: 1950)*.

[72] Chung SW, Park JW, Lee SA, Eo SK, Kim K (2010). Thrombin promotes proinflammatory phenotype in human vascular smooth muscle cell. *Biochemical and Biophysical Research Communications*.

[73] Kobiyama K, Ley K (2018). Atherosclerosis. *Circulation Research*.

[74] Libby P (2021). The changing landscape of atherosclerosis. *Nature*.

[75] Borén J, Chapman MJ, Krauss RM, Packard CJ, Bentzon JF, Binder CJ (2020). Low-density lipoproteins cause atherosclerotic cardiovascular disease: pathophysiological, genetic, and therapeutic insights: a consensus statement from the European Atherosclerosis Society Consensus Panel. *European Heart Journal*.

[76] Haley KJ, Lilly CM, Yang JH, Feng Y, Kennedy SP, Turi TG (2000). Overexpression of eotaxin and the CCR3 receptor in human atherosclerosis: using genomic technology to identify a potential novel pathway of vascular inflammation. *Circulation*.

[77] Economou E, Tousoulis D, Katinioti A, Stefanadis C, Trikas A, Pitsavos C (2001). Chemokines in patients with ischaemic heart disease and the effect of coronary angioplasty. *International Journal of Cardiology*.

[78] Ardigo D, Assimes TL, Fortmann SP, Go AS, Hlatky M, Hytopoulos E (2007). Circulating chemokines accurately identify individuals with clinically significant atherosclerotic heart disease. *Physiological Genomics*.

[79] Cross DS, McCarty CA, Hytopoulos E, Beggs M, Nolan N, Harrington DS (2012). Coronary risk assessment among intermediate risk patients using a clinical and biomarker based algorithm developed and validated in two population cohorts. *Current Medical Research and Opinion*.

[80] Younus M, Fan W, Harrington DS, Wong ND (2019). Usefulness of a Coronary Artery Disease Predictive Algorithm to Predict Global Risk for Cardiovascular Disease and Acute Coronary Syndrome. *The American Journal of Cardiology*.

[81] Wu JM, Hsieh TC, Yang CJ, Olson SC (2013). Resveratrol and its metabolites modulate cytokine-mediated induction of eotaxin-1 in human pulmonary artery endothelial cells. *Annals of the New York Academy of Sciences*.

[82] Saleh M, Ambrose JA (2018). Understanding myocardial infarction. *F1000Research*.

[83] Thygesen K, Alpert JS, White HD, Joint ESC/ACCF/AHA/WHF Task Force for the Redefinition of Myocardial Infarction (2007). Universal definition of myocardial infarction. *Journal of the American College of Cardiology*.

[84] Ambrose JA, Najafi A (2018). Strategies for the Prevention of Coronary Artery Disease Complications: Can We Do Better?. *The American Journal of Medicine*.

[85] Zee RYL, Cook NR, Cheng S, Erlich HA, Lindpaintner K, Ridker PM (2006). Multi-locus candidate gene polymorphisms and risk of myocardial infarction: a population-based, prospective genetic analysis. *Journal of Thrombosis and Haemostasis: JTH*.

[86] Kalayci M, Gul E (2022). Eotaxin-1 Levels in Patients with Myocardial Infarction. *Clinical Laboratory*.

[87] Bonaros N, Sondermeijer H, Schuster M, Rauf R, Wang SF, Seki T (2008). CCR3- and CXCR4-mediated interactions regulate migration of CD34+ human bone marrow progenitors to ischemic myocardium and subsequent tissue repair. *The Journal of Thoracic and Cardiovascular Surgery*.

[88] Yuan D, Tie J, Xu Z, Liu G, Ge X, Wang Z (2019). Dynamic Profile of CD4+ T-Cell-Associated Cytokines/Chemokines following Murine Myocardial Infarction/Reperfusion. *Mediators of Inflammation*.

[89] Lu Q, Zhang Y, Geng T, Yang K, Guo K, Min X (2022). Association of Lifestyle Factors and Antihypertensive Medication Use With Risk of All-Cause and Cause-Specific Mortality Among Adults With Hypertension in China. *JAMA Network Open*.

[90] Carey RM, Moran AE, Whelton PK (2022). Treatment of Hypertension: A Review. *JAMA*.

[91] Mills KT, Stefanescu A, He J (2020). The global epidemiology of hypertension. *Nature Reviews. Nephrology*.

[92] Liang C, Ni G, Ma J, Liu H, Mao Z, Sun H (2017). Impact of Tag Single Nucleotide Polymorphisms (SNPs) in CCL11 Gene on Risk of Subtypes of Ischemic Stroke in Xinjiang Han Populations. *Medical Science Monitor: International Medical Journal of Experimental and Clinical Research*.

[93] Varghese B, Joy CA, Josyula JVN, Jangili S, Talukdar RK, Mutheneni SR (2023). Machine learning-based protein signatures for differentiating hypertensive disorders of pregnancy. *Hypertension Research: Official Journal of the Japanese Society of Hypertension*.

[94] Hart PMB, Stephenson NL, Scime NV, Tough SC, Slater DM, Chaput KH (2022). Second trimester cytokine profiles associated with gestational diabetes and hypertensive disorders of pregnancy. *PloS One*.

[95] McCarthy CP, Shrestha S, Ibrahim N, van Kimmenade RRJ, Gaggin HK, Mukai R (2019). Performance of a clinical/proteomic panel to predict obstructive peripheral artery disease in patients with and without diabetes mellitus. *Open Heart*.

[96] McCarthy CP, Ibrahim NE, van Kimmenade RRJ, Gaggin HK, Simon ML, Gandhi P (2018). A clinical and proteomics approach to predict the presence of obstructive peripheral arterial disease: From the Catheter Sampled Blood Archive in Cardiovascular Diseases (CASABLANCA) Study. *Clinical Cardiology*.

[97] Mosedale DE, Smith DJ, Aitken S, Schofield PM, Clarke SC, McNab D (2005). Circulating levels of MCP-1 and eotaxin are not associated with presence of atherosclerosis or previous myocardial infarction. *Atherosclerosis*.

[98] Nazarinia D, Behzadifard M, Gholampour J, Karimi R, Gholampour M (2022). Eotaxin-1 (CCL11) in neuroinflammatory disorders and possible role in COVID-19 neurologic complications. *Acta Neurologica Belgica*.

[99] Ivanovska M, Abdi Z, Murdjeva M, Macedo D, Maes A, Maes M (2020). CCL-11 or Eotaxin-1: An Immune Marker for Ageing and Accelerated Ageing in Neuro-Psychiatric Disorders. *Pharmaceuticals (Basel, Switzerland)*.

[100] Scabia G, Testa G, Scali M, Del Turco S, Desiato G, Berardi N (2021). Reduced ccl11/eotaxin mediates the beneficial effects of environmental stimulation on the aged hippocampus. *Brain, Behavior, and Immunity*.

